# Treatment of auricular relapsing polychondritis in a Saudi child using only non-steroidal anti-inflammatory drugs: a case report

**DOI:** 10.11604/pamj.2020.37.217.22998

**Published:** 2020-11-04

**Authors:** Ashwaq Ahmed AlE'ed

**Affiliations:** 1 Department of Pediatrics, College of Medicine, Qassim University, Al-Qassim, Saudi Arabia

**Keywords:** Polychondritis, autoimmune disease, NSAID, auricular, case report, Saudi Arabia

## Abstract

Relapsing polychondritis (RP) is an autoimmune disease that can involve multiple sites within the human body. It is characterized by recurrent bouts of painful cartilage inflammation, and it can cause severe complications if it affects the vital organs. This report describes the case of a five-year-old child with limited auricular RP. The patient's history was obtained from his family, and a physical examination was performed at a pediatric rheumatology clinic. The patient was successfully treated using only a non-steroidal anti-inflammatory drug, and he completely recovered. This treatment and recovery have not been reported in the literature. Therefore, these results are worthy of mention in order to avoid the use of immunosuppressant medications with localized involvement.

## Introduction

Relapsing polychondritis (RP) is an autoimmune disease that was first described in 1923, it is a progressive, destructive inflammatory disease that can affect multiple sites within the human body, commonly affects adults but can also, more rarely, occur in children [1]. Several case reports and studies have described different presentations and treatment modalities associated with childhood RP [1,2]. Severe secondary complications may occur from airway involvement, which is more likely among children than adults [3]. Additionally, RP reviews and reports have shown that ethnicity may play a role in the disease´s severity and age of onset [2]. A recent review summarized more than 40 case reports and studies on RP, showing the reliability of treatment regimens, including immunosuppressant medications and even surgical interventions, but no well-established guidelines exist to treat pediatric patients with RP [1]. Thus, this case report describes a rare case of RP with limited auricular involvement that was treated using only a non-steroidal anti-inflammatory drug (NSAID).

## Patient and observation

A previously disease-free five-year-old boy presented with a three-week history of redness, swelling, and ulceration of the left earlobe. An ulcer had developed at the helix and lobule of the ear a few days after symptom onset. The patient was treated using topical corticosteroids, and the size of the ulcer improved; however, the redness and pain persisted. After three months, lesions started to appear on his right ear, exhibiting the same painful redness and ulceration. Once again, topical steroids were prescribed, but the response was minimal and slow for both ears. As the lesions persisted and no improvement followed the use of oral and topical antibiotics or topical steroids, the patient was seen by a dermatologist and then referred to a pediatric rheumatologist. The patient had no history of fever, skin rash, preceding infection, trauma, or food or drug allergies. He had no respiratory or musculoskeletal symptoms and, upon other systemic reviews, was found to be medically unremarkable. His family and social history were negative for any genetic or autoimmune disease.

**Figure 1:**
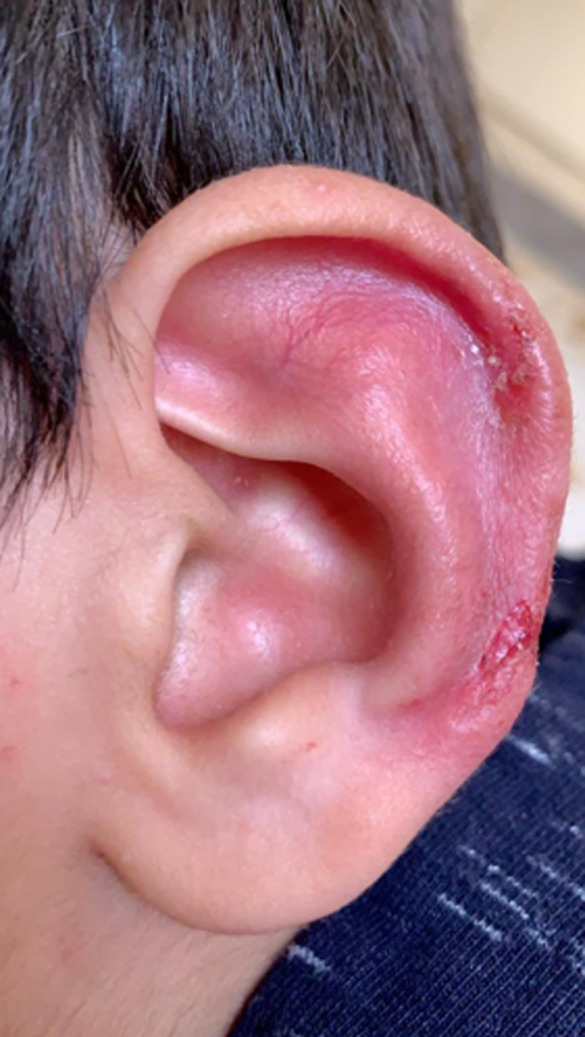
RP lesions at presentation

**Figure 2:**
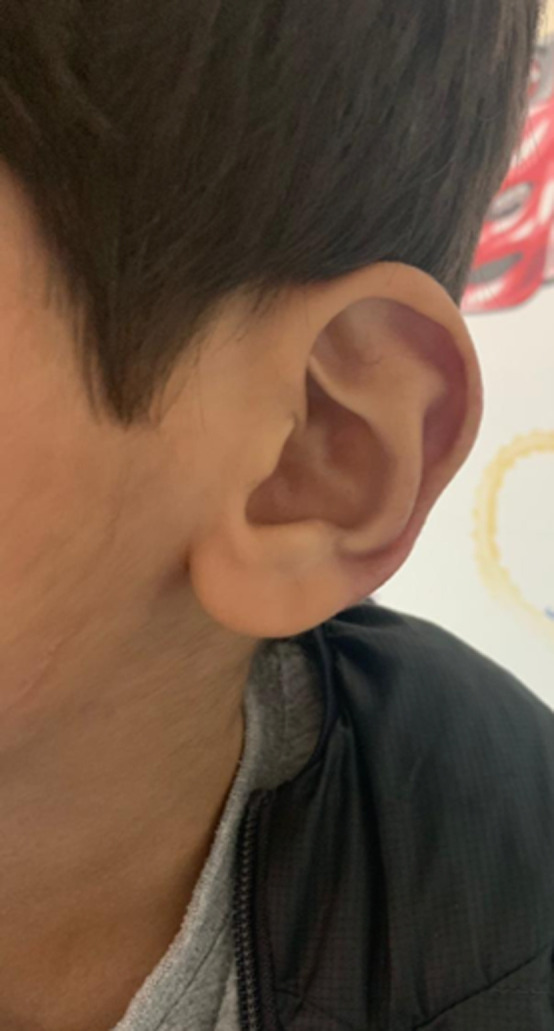
significant improvements in the same ear after treatment

The clinical findings at the time of evaluation by the pediatric rheumatologist indicated that the patient was looking well, an alert child with a good body build, afebrile with normal vital signs. Upon local examination of his ear, nose, and throat (ENT), significant redness and erosive wounds were found in both ears, though more on the left side (Figure 1). The ears were tender, and the rest of the ENT examination was normal. Cardiovascular, respiratory, gastrointestinal, musculoskeletal, and central nervous system examinations were clinically normal. Laboratory examinations revealed a white blood cell count of 8.95 x 109/L (normal), an erythrocyte sedimentation rate of 32 (slightly elevated), negative C-reactive protein, negative antinuclear antibody, negative leishmania antibody immunoglobulin M, and complement within normal ranges. The antibodies of Type II collagen were not available at our center. A chest computerized tomography (CT) scan was done to rule out respiratory tract involvements, and the results were normal. The patient was screened by the otorhinolaryngology and ophthalmology departments for the involvement of other sites, and these findings were normal. Also, the joint and cardiac evaluations via echocardiogram were negative. Treatment was discussed with the patient´s family, and they refused a corticosteroid, so the patient was started on an NSAID (10 mg/kg of ibuprofen every eight hours for six weeks). The patient showed improvement from the fourth week of treatment, with healing of the lesions and disappearance of the redness along with minimal remanence. After six weeks of treatment, he completely recovered (Figure 2), and all inflammatory markers-mainly ESR-normalized.

## Discussion

Relapsing polychondritis is a rare disease that mainly affects adults; childhood-onset RP represents only 5-10% of all reported cases [1,2]. The etiology of RP remains unknown, although it likely involves an autoimmune mechanism against collagen Type II [2]. RP is a systemic disease that affects the cartilage throughout the body, and it leads to inflammation and the destruction of various cartilaginous structures, including the ears, nose, larynx, trachea, bronchi, peripheral joints, eyes, heart, and skin [4]. The age of onset in children varies between 1 and 17 years, with cases below 2 years rarely reported [5]. Since RP is very rare in children, its diagnosis can be delayed by up to five years [6]. Referral to a pediatric rheumatologist can also take up to three years [2]. Biopsy and antibody evaluations are generally inconclusive, making RP challenging to diagnose [1,6]. Furthermore, unlike adult RP, pediatric-onset RP is rarely associated with other autoimmune diseases [1]. The progressive nature of RP may lead to severe complications, including hearing loss, vertigo, and saddle nose, affecting 26%, 13%, and 29% of patients, respectively (adult report) [4]. However, the mortality and morbidity of RP are related to cardiac and respiratory complications [1].

Auricular chondritis is the most common manifestation of RP, affecting up to 90% of patients with the disease, followed by nasal, laryngeal, and articular involvement [3,7]. The disease usually involves the cartilaginous part of the ear and causes redness (sometimes dark discoloration), pain, and tenderness. RP typically spares the non-cartilaginous auricular lobe, as was the case with the present patient. However, the cartilaginous parts of both the patient´s ears were affected. Other diseases were excluded through differential diagnoses, including infectious and autoimmune diseases that did not fit RP diagnostic criteria [2,8], as RP can be a presentation of other autoimmune diseases [1]. Due to the rarity of pediatric-onset RP, its prevalence remains unknown, and validated guidelines for treatment do not exist. Various medications have been used in previously reported cases and studies, including non-steroidal anti-inflammatory drugs (NSAIDs), corticosteroids, hydroxychloroquine [1], colchicine, dapsone, and disease-modifying anti-rheumatic drugs (DMARDs) [1,2]. Indeed, NSAIDs can be effective against certain forms of RP, such as the non-severe forms of auricular and nasal chondrites and arthritis [1,9]. One report concerned a patient treated with anti-TNF therapy in addition to DMARDs; this treatment resulted in significant improvement and, ultimately, resolution of the disease [1,10]. The present patient was treated using only an NSAID (ibuprofen); he completely recovered, and he remained symptom-free more than six months after ceasing treatment. Further evaluations by the otorhinolaryngology and ophthalmology departments were normal.

## Conclusion

In conclusion, pediatric-onset RP is challenging to diagnose and involves a broad spectrum of treatments. This report presents a case of isolated RP that responded well to NSAIDs alone, with complete recovery. A further study is suggested to improve the approach for patients with this disease and to establish guidelines for treatment.
